# International study opportunities in the dentistry degree programme at the University of Münster – a needs assessment of student interest and demand

**DOI:** 10.3205/zma001757

**Published:** 2025-06-16

**Authors:** Julian Hettkamp, Jan C. Becker, Sönke Scherzer, Bernhard Marschall, Benjamin Ehmke, Petra Scheutzel, Anna Junga

**Affiliations:** 1 University of Münster, Faculty of Medicine, Polyclinic for Periodontology and Tooth Preservation, Münster, Germany; 2 University of Münster, Faculty of Medicine, Institute for Education and Student Affairs, Münster, Germany; 3 University of Münster, Faculty of Medicine, Polyclinic for Prosthetic Dentistry and Biomaterials, Münster, Germany

**Keywords:** dentistry, ERASMUS, stay abroad, internationalisation

## Abstract

**Introduction:**

Stays abroad in the form of study visits and clinical traineeships, for example, have been rare in dentistry degree programmes in Germany to date. The new ZApprO (date of issue: 8 July 2019) offers the opportunity to integrate stays abroad into the degree programme through the introduction of modules, ECTS points and clinical traineeships, among other things. The aim of this study was to analyse the student perspective on this topic.

**Methodology:**

In the summer term of 2021, a voluntary and anonymous online survey was conducted among dental students at the University of Münster. In addition to demographic data, the questionnaire also included key questions on content.

**Results:**

With a response rate of 55%, a total of 371 students took part in the survey. 96% of the study participants stated that they would like to gain experience abroad as part of their studies, almost half of them even in the case of missing or only partial recognition of academic achievements. This includes clinical traineeships (30%) and semester-long stays (32%) or both (28%). The loss of time in the progression of studies, financing as well as time-consuming planning and family obligations are the most frequently cited reasons against a stay abroad.

**Conclusion:**

The survey shows a very high level of interest among dental students in university-supported stays abroad. This includes both shorter periods of time, such as clinical traineeships, as well as longer stays, e.g. as part of Erasmus collaboration programmes. Based on the results, university-supported stays abroad should be made possible.

## Zusammenfassung

**Einleitung:**

Auslandsaufenthalte in Form von z.B. Studienaufenthalten und Famulaturen, sind im Studiengang Zahnmedizin in Deutschland bisher selten. Die neue ZApprO (Ausfertigungsdatum: 08.07.2019) bietet u.a. durch Einführung von Modulen, ECTS-Punkten und Famulaturen die Möglichkeit Auslandsaufenthalte in das Studium zu integrieren. Ziel der vorliegenden Studie war es, zwecks einer gezielten Vorbereitung die studentische Sichtweise dazu exemplarisch zu erheben.

**Methodik:**

Im Sommersemester 2021 wurde eine freiwillige und anonyme online Umfrage unter den Studierenden der Zahnmedizin am Studienstandort Münster durchgeführt. Der Fragebogen umfasste neben demographischen Daten auch inhaltliche Schwerpunktfragen.

**Ergebnisse:**

Entsprechend einer Rücklaufquote von 55%, haben insgesamt 371 Studierende an der Umfrage teilgenommen. Dabei gaben 96% der Studienteilnehmer*innen an, im Rahmen ihres Studiums Auslandserfahrung sammeln zu wollen, knapp die Hälfte davon sogar im Fall ausbleibender oder lediglich partieller Anerkennung von Studienleistungen. In Frage kommen Famulatur (30%) - und semesterumfassende Aufenthalte (32%) zu nahezu gleichen Teilen, weitere 28% würden beide Optionen in Betracht ziehen. Der Zeitverlust im Studienfortschritt, die Finanzierung sowie aufwendige Planung und familiäre Verpflichtungen sind die am häufigsten genannten Gründe gegen einen Auslandsaufenthalt.

**Schlussfolgerung:**

Die Umfrage belegt ein sehr hohes Interesse von Studierenden der Zahnmedizin an universitär geförderten Auslandsaufenthalten. Dabei kommen sowohl kürzere Zeitfenster, wie etwa für Famulaturen infrage, aber auch längere Aufenthalte wie z.B. im Rahmen von Erasmus-Kooperationen. Auf Basis der Ergebnisse sollte die Etablierung von universitär geförderten Auslandsaufenthalten ermöglicht werden.

## Introduction

In view of increasing globalisation and internationalisation, the exchange of scientific knowledge and experience as well as interdisciplinary networking in an academic context is becoming increasingly important. In 1987 the European Union decided to create the basis for a funding programme that promotes exchange and understanding in a peaceful Europe [https://www.erasmusplus.de/wer-wir-sind/30-jahre-erasmus]. “ERASMUS” stands for “European Action Scheme for the Mobility of University Students” and describes a measure to promote, finance and organise academic exchanges between different European countries [1]. Specifically, students in this programme can use established structures and financial support to gain experience in another European country as part of a semester or internship abroad. To date, around 10 million Europeans have benefited from the ERASMUS programme [https://www.erasmusplus.de/wer-wir-sind/30-jahre-erasmus].

The reasons for stays abroad are manifold and have already been researched in various studies: the further development of professional and personal skills, learning or improving a foreign language, improving one’s own career opportunities, but also cultural exchange are cited as the most important aspects for the decision to spend an “ERASMUS semester” [2], [3], [4]. As Zebryk et al. 2021 showed, the majority of ERASMUS alumni were able to positively change their learning approaches and strategies as a result of the exchange, which meant that students gained not only culturally but also personally [5]. The demands of a semester abroad are not comparable with other degree programmes in terms of practical elements such as patient treatment and require special precautions and capacities. International research, the exchange between different teaching formats and especially the interdisciplinary treatment approach is of particular importance to dental students for their studies, but also for their future careers. 

The ERASMUS exchange programme in medicine, in contrast to dentistry, was established at the Faculty of Medicine at the University of Münster (UM) back in the early 1990s. According to an unpublished study by UM, in which all dental faculties in Germany were asked to participate, the reasons for the lack of exchange opportunities in dentistry are in particular the sometimes large differences between the curricula and bureaucratic hurdles between the faculties. The new version of the ZApprO, which has been in force since 2019, now offers the necessary prerequisites for integrating stays abroad into the degree programme through the introduction of modules, ECTS points and clinical traineeships, among other things (§15 Absatz 5 ZAppro). 

The aim of this study was to evaluate the needs of the student body at the study location Münster in order to start a corresponding establishment process based on this. Furthermore, key points are to be identified in order to be able to take the interests and needs of the student body into account during implementation and to address possible difficulties directly.

## Methods

The “dentistry exchange programme” questionnaire was compiled in an iterative process. The aim was to gain an overview of the prevailing mood in the student body in order to evaluate the usefulness of the process for developing international cooperation programmes on this basis. To this end, various topics, such as general interest in studying abroad, language skills, but also aspects such as funding, were surveyed and analysed using an online questionnaire. Students from all semesters at the University of Münster Dentistry programme were asked about their individual attitudes towards ERASMUS semesters abroad and clinical placements abroad.

The questionnaire was developed at the Institute for Education and Student Affairs (IfAS, authors AJ and JCB) of the Medical Faculty of Münster in cooperation with the Dental Student Council (author JH). The possible questions were collected in a pool of questions and submitted to the International Office (Faculty of Medicine) and the clinic directors (including the study programme coordinator) for coordination in addition to the above-mentioned participants. The final questionnaire was created by consensus. The final version was presented to the student council as a test group to check for formal and content-related errors. The participants were not involved in the design of the questionnaire. After a final review, the questionnaire was released for publication.

The questionnaire consists of twelve items with an open and closed response format (see attachment 1 ). Free text questions were deliberately included in order to capture individual opinions such as wishes and fears in addition to the descriptive data. The first three questions are based on demographic aspects such as gender, age and semester of study. In order to capture the basic mood of the students, the following block of questions collects data on the general interest in studying abroad, the creditability of academic achievements, as well as preferences regarding the length and format of the stay abroad. In the last block, questions are asked about language skills, preferred cooperation partners, reasons against a semester abroad and the maximum budget available. The aim was to gain as differentiated a picture as possible of needs, interest and, in particular, language skills in order to begin the process of finding possible cooperation partners. Students from all preclinical and clinical semesters of dentistry at the Münster site were selected as study participants (see table 1 (Tab. 1)).

At the time of publication of the questionnaire, this amounted to 674 people. The students were contacted via the e-mail distribution list of the respective semesters and asked to take part in this survey. They were specifically informed of the voluntary and anonymous nature of this survey, as well as the possibility of cancelling the data sent. Furthermore, students did not suffer any disadvantages if they did not take part in the questionnaire. 

The data collection and collation took place over a period of 3 months between July and September 2021. The questionnaire was conducted via the online tool LIME-Survey (Version 5.0.0; LimeSurvey GmbH, Hamburg, Germany). This website is a secure website that complies with the UM's data protection requirements for scientific questionnaires and is used as standard. Participation in the survey was (exclusively) possible via the link sent. 

The data collected was analysed using the SPSS programme (version 29.0.0.0. (241); IBM, Armonk, USA). Figures and graphics were created using SPSS, MS Excel (version Excel 2405; Microsoft Corporation, USA) and R (version 4.4.4.1; R Core Team, USA) (see attachment 2 ).

## Results

A total of 371 students took part in the survey, which corresponds to a response rate of 55.04%. The gender ratio corresponded to the population of the dental medicine degree programme in Germany [6], with 253 women (73.12%), 92 men (26.58%) and 1 diverse person (0.29%). The average age of all participants was 22.17 years (SD±3.596) with an age range between 17 and 40 years. A gender differentiation showed that men were on average almost one year older at 22.72 years (SD±4.1) compared to women at 21.97 years (SD±3.4). This is also in line with the population of all students in Germany [7].

The participants were predominantly between the 1^st^ and 10^th^ semester, with the maximum (two participants) being in their 20^th^ semester. It is striking that the participation rate of preclinical students (preclinical participation rate: 54.80%) was almost twice as high as that of clinical students (clinical participation rate: 33.20%).

**Figure 1:**
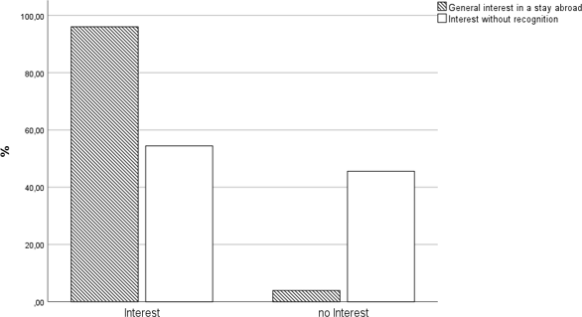
Interest in stays abroad with and without recognition of credits; interest in general n=316, interest without recognition of credits n=166, no interest in general n=13, no interest without recognition of credits n=139

96.04% of survey participants (316 students) stated that they would generally like to gain experience abroad as part of their degree programme. Half of all participants (54.43%) would even be willing to take part in a stay abroad if their studies were only partially completed, or even not be recognised at all (see figure 1 (Fig. 1)). There was no significant difference between the genders (men: 56.32%, women 47.7%). 

When asked about the preferred type of stay abroad, there was no clear tendency in favour of shorter (usually lasting a few weeks) clinical traineeships (29.65%) or longer (usually semester-long) Erasmus stays (32.08%). Both types of stays abroad were almost equally popular, with a quarter of respondents (27.49%) expressing interest in both options.

When asked about the best time to go abroad, respondents were able to give multiple answers regarding their preference (seventh, eighth or ninth semester). The possible dates were preselected depending on the curriculum and the experience level of the students. Based on this option, 51.22% of participants named several points in time. If the sum of all answers given is analysed, the eighth semester (41.04%) is the most popular, closely followed by the seventh semester (36.71%). Only the ninth semester appears to be of less interest with 22.24%. 

**Figure 2:**
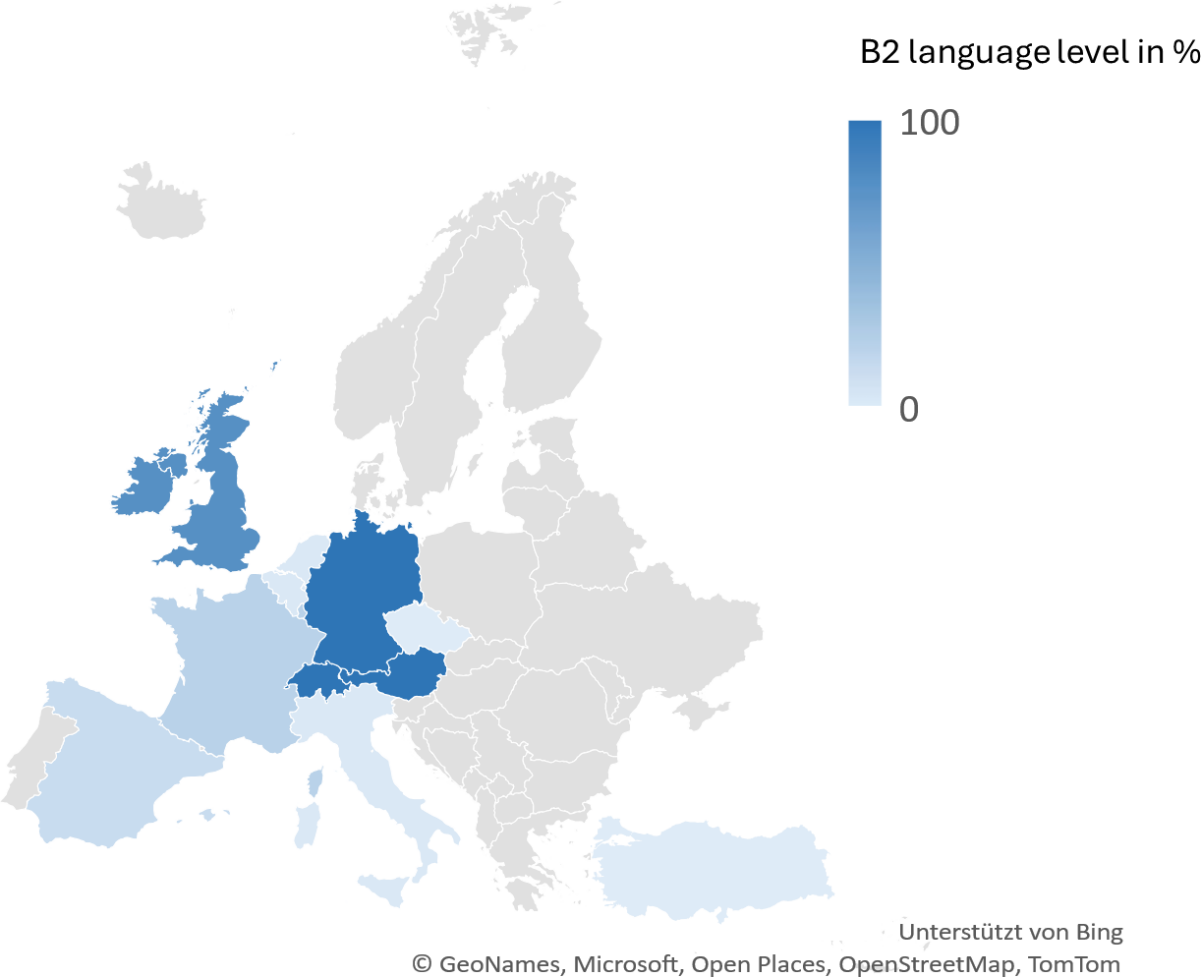
Map diagram of Europe plus Turkey, representation of existing language skills at B2 level in % of respondents, n=436, created with Microsoft^®^ Excel^®^ for Microsoft 365 MSO (Version 2408 Build 16.0.17928.20114)

The students were also asked about their previous language skills. Language certificates at level B2 or higher are primarily documented in English (85.89%), French (23.72%) and Spanish (13.51%). In addition, a small proportion of participants stated that they had acquired a corresponding language certificate for Italian, Turkish (2.7% each) or Dutch (2.4%) (see figure 2 (Fig. 2)).

A larger selection of 13 possible partner universities, which were positively requested in advance by the International Office with regard to possible cooperation, were asked in the questionnaire with the option of multiple selection. Oulu, Finland (59.19%), Basel, Switzerland (58.26%), Padua, Italy (57.01%) and Coimbra, Portugal (49.84%) showed significant approval as direct co-operations. In addition, the USA (76.01%), Spain (66.98%), France (54.83%) and Japan (50.15%) were named as possible destinations by a large number of participants (multiple selection possible).

Multiple answers were also possible to the question of possible specialisations. The analysis of all responses revealed a comparable level of interest among students in all specialist departments. Operative dentistry (KONS) (27.17%) was followed by orthodontics (KFO) (24.77%), oral and maxillofacial surgery (MKG) (24.43%) and finally prosthetics (23.63%). 34.77% of respondents stated that they were equally interested in all subjects.

**Figure 3:**
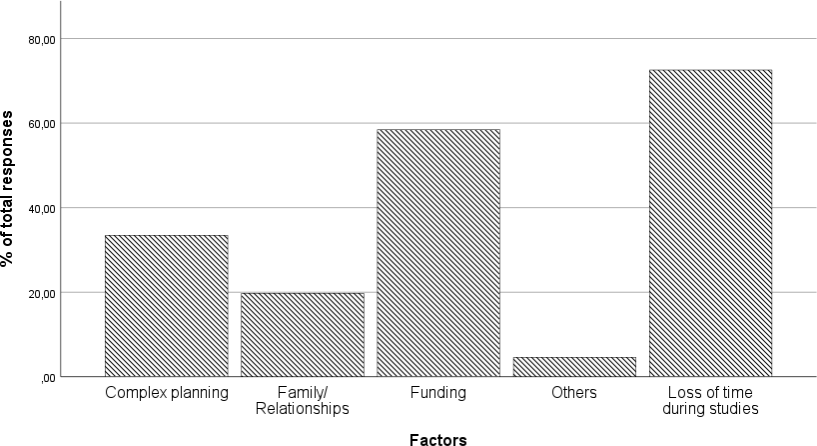
Cited factors against a stay abroad in % of total responses; time-consuming planning n=95, family/relationship n=56, financing n=166, other n=13, loss of study time n=206, multiple answers possible

In the following section (see figure 3 (Fig. 3)), students were asked about the difficulties they see in relation to a stay abroad. In addition to the loss of time during their studies (65.81%), financing (53.04%), concerns about time-consuming planning (30.35%) and family reasons (17.89%) were named as factors that would prevent students from going abroad. Students were given the opportunity to give short answers. Comments such as “non-recognised achievements and the associated loss of time would be a heavy burden, both financially and socially, as the cohort would be changed”, “uncertainty about financing if you have to finance everything yourself and it is not clear in advance what costs you will incur” or “exceeding the standard period of study” were frequently mentioned (see attachment 3 ).

**Figure 4:**
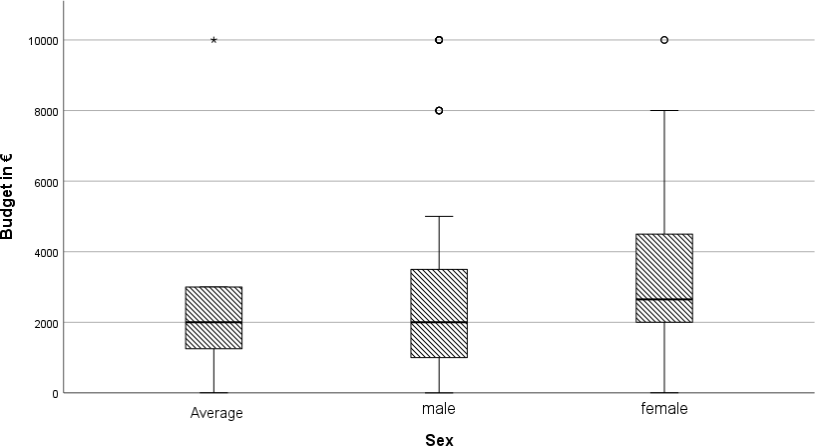
Boxplot, budget in € for stays abroad, total and broken down by gender; total n=223, male n=68, female n=155

With regard to funding, students were also asked what maximum budget they would spend on a stay abroad. On average, this was stated as €2929.14 (SD± €3296.23). At €3365.03 (SD±€2835.98), women are prepared to invest significantly more money on average than men at €2599.8 (SD±€2321.36) (see figure 4 (Fig. 4)).

## Discussion

The aim of the study was to record the interest and general conditions of students with regard to stays abroad in order to enable well-founded measures to expand the corresponding programmes in the future on the basis of the results. Based on the response rate and the gender ratio, it can be assumed that the survey is representative, at least for Münster as a study location. 

In principle, this study was able to show that there is a high level of interest in university stays abroad when studying dentistry. The interest is so pronounced that half of the respondents would be prepared to complete a period abroad, even if this would not contribute directly to the progression of their studies due to a lack of credit transfer. Based on the assumption that primarily interested students took part in the survey, stays abroad are primarily of interest to students in the pre-clinical section. 

The seventh and eighth semesters were increasingly cited as the ideal time. Overall, both shorter time frames (e.g. clinical traineeships) and longer stays, such as Erasmus collaborations, appear to be of interest to students. For a successful establishment of a stay abroad, the survey revealed several points that need to be further elaborated by the faculty.

One of the most important aspects to be considered here is capacity. Assuming that all students who did not take part in the survey are not interested in studying abroad, this still leaves a large number of interested students (55.04% overall). With an average programme size of 55 students per semester and a limited possible time window (due to state examinations and preparation time), the demand per semester is very high. 

Supervising a larger number of foreign students, as is possible in human medicine due to the lower proportion of one-to-one training close to the patient (e.g. treatment courses), is not practicable in dentistry. Moreover, an exchange can only be realised if students use the capacities of cooperating faculties to roughly the same extent. This implies that there must be a limited selection of fixed cooperation partners in order to enable an exchange within this fixed framework. A conceivable number would be three selectable universities and a total capacity per winter or summer term of a maximum of three to five students. This would correspond to 10% of a semester cohort. This does not yet consider further offers in the area of clinical traineeships (“Famulatur”) abroad, which would create additional capacity.

Since language is essential as a decisive criterion for the success of a possible cooperation and learning and improving one’s own language skills is an extremely relevant criterion for 90% of students when deciding where to study [5], this aspect must be given appropriate importance. Zebryk et al. showed that 94% of students reported an improvement in their professional language skills in the language of the host country, with 77% even rating this as particularly high [5]. In the survey, universities in Finland, Switzerland and Italy were mentioned most frequently as possible partners. However, if this is seen in the context of the language skills reported by Münster students, it would make sense to choose primarily English-speaking (and secondarily Spanish and French-speaking) universities, even if other languages are spoken in individual cases. Destinations such as Finland or Japan are certainly culturally attractive [8] but can only be meaningfully included if participation in the events is possible in English. 

As the aim is bilateral cooperation, it should also be noted that the University of Münster only offers courses in German. In medicine, a language level of B2 is a prerequisite for applying for a semester abroad in the respective national language.

Furthermore, the content-related specialisation of a possible cooperation university is an important aspect in the decision-making process. In order to do justice to the balanced interest in all specialist disciplines (oral and maxillofacial surgery, orthodontics, prosthetics and operative dentistry), the selected universities should be chosen on the basis of a sound range of courses in these areas. 

A known good education in the above-mentioned areas is particularly relevant, as according to Marinescu et. al, the reputation of the university is becoming increasingly important for students in advanced studies when making a decision [8]. A corresponding expertise of the respective departments could promote the interest of our students accordingly and suggests a positive effect with regard to the professionalisation of their own knowledge and individual learning success. Zebryk et al. have already described the positive changes to be expected with regard to the learning approach in clinical practice and evidence-based medicine [5]. 

Despite all these positive aspects, the financing of an exchange and the unavoidable loss of time for students is of great importance. Previous studies have already analysed the influence of funding in detail and attempted to define its role in the decision-making process [9]. Dental students sometimes have high financial expenses for material purchases, which can be between €1000 and €10,000 depending on the study location [10]. In order not to exclude students from an experience abroad for monetary reasons and thus discriminate against socially disadvantaged students in particular, financial support options are required [11]. In addition to the aspect discussed above, the choice of location for a semester abroad can also depend on the expected costs and available funding programmes [12]. Programmes such as ERASMUS (European exchange) or PROMOS (worldwide exchange) support the financing of tuition fees and accommodation through grants, but sometimes cannot cover all costs incurred. In order to increase the participation of disadvantaged and hard-to-reach groups in international projects, additional financial resources have been made available by the EU and the federal government so that improved support is possible [13]. 

Furthermore, 66% of the study participants stated that the loss of time during their studies was one of the biggest obstacles to a stay abroad. Compared to other degree programmes such as foreign language subjects in teacher training [14] or business administration [15], where curricular and extracurricular stays abroad are already firmly anchored, a possible loss of time seems to be a primary problem in dentistry. However, through an adapted study plan with selected cooperation partners, the faculty should set the goal of increasingly recognising achievements in order to minimise any loss of time. It should also be discussed whether a semester abroad could also be carried out in the pre-clinical section (e.g. 5^th^ or 6^th^ semester). The language barrier is negligible here and it is possible to achieve a comparable level of competence through standardised training in phantom courses. A disadvantage is the close implementation between two state examinations (4^th^ and 6^th^ semester) and the lack of patient contact, which is relevant for personal development. If potential partner universities prefer a module in these semesters, this should be assessed individually.

Once a semester-long ERASMUS stay abroad has been established, the possibility of clinical traineeships abroad should also be considered. Within the framework of the new ZAppro, clinical traineeships abroad can also be recognised in the curriculum. Programmes such as PROMOS can be used to finance traineeships abroad from a certain minimum duration. The advantage of this programme is the individuality of location and duration. Despite its broad-based funding, ERASMUS is subject to strict regulations (internal capacity limits, minimum stay of three months, etc.) so that clinical traineeships abroad should be established as an additional option. 

The analysis of all the data collected emphasises the clear vote of the student body to establish stays abroad as part of the study of dentistry at the University of Münster. Nevertheless, with a response rate of 55%, a certain selection bias cannot be ruled out, which must be taken into account as a limitation of the study. In addition, a further distortion of the results cannot be ruled out due to the voluntary nature of this survey. Even if more comparable study conditions prevail at the locations as a result of the new licencing regulations, certain differences in the study plan may mean that the positive results cannot be applied to other locations. Further studies with extended samples, also at other study locations in Germany, should be carried out to investigate the topic. The effect of a clinical traineeship abroad could also be compared with a clinical traineeship in Germany. 

The advantages in terms of language development, changes in learning strategy and knowledge acquisition clearly outweigh the disadvantages [16]. According to Bryla et al., a third of all ERASMUS students state that experience abroad had a significant impact on their personal fulfilment, academic development and professional position. The current National Education Report also states that the benefits for participants and institutions in the ERASMUS+ programme are very great compared to those who do not participate and that these results will continue in the long term [13].

The new ZApprO now also offers the necessary framework conditions for this, so that the recognition of academic achievements can be guaranteed if they are successfully completed abroad. The financial hurdles mentioned could also be reduced, for example through cooperation within the framework of the EU’s ERASMUS project and thus financial support for students.

## Conclusion

The high level of interest shown here by dental students in stays abroad should be interpreted as a clear mandate to facilitate stays abroad on the basis of the new version of the ZApprO. 

In order to ensure planning and creditability for both students and the university, fixed co-operations with partners in English-, Spanish- or French-speaking EU countries and, within the framework of PROMOS, also worldwide, should be found. One limitation in the search for suitable partners, however, is that the courses offered at UM’s Faculty of Medicine are exclusively in German. The range of disciplines on offer and the reputation of teaching at the university locations should also play a role in the selection process. The expected costs for students can be significantly reduced by using funding programmes such as the ERASMUS programme. Based on the given requirements, a semester-long exchange could be realised for a relevant proportion of students. In addition, clinical traineeships could be organised more freely and individually (language, culture) for several weeks.

## Authors’ ORCIDs

- Julian Hettkamp: [0009-0000-7481-6115]
- Sönke Scherzer: [0000-0002-7197-2101]
- Bernhard Marschall: [0000-0002-1354-8687]
- Benjamin Ehmke: [0000-0002-2418-6765]
- Anna Junga: [0000-0002-4165-9114]

## Competing interests

The authors declare that they have no competing interests. 

## Supplementary Material

Questionnaire “Survey exchange programme dentistry”

Overall evaluation of the “dentistry exchange survey”

Individual responses to the question: What factors would prevent you from going abroad?

## Figures and Tables

**Table 1:**
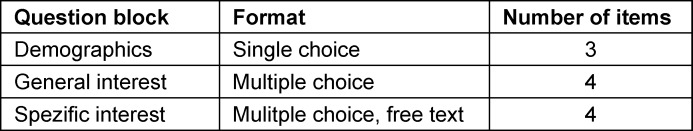
Structure of the questionnaire
