# Supplementary material for: International study opportunities in the dentistry degree programme at the University of Münster – a needs assessment of student interest and demand
Source: GMS J Med Educ. 2025 Jun 16;42(3):Doc33. doi: 10.3205/zma001757 (PMC12286880; doi:10.3205/zma001757)
Supplement: Questionnaire “Survey exchange programme dentistry” [file JME-42-33-s-001.pdf]

Attachment 1: Questionnaire “Survey exchange programme dentistry”

Attachment 1 to Hettkamp J, Becker JC, Scherzer S, Marschall B, Ehmke B, Scheutzel P, Junga A. *International study opportunities in the dentistry degree programme at the University of Münster – a needs assessment of student interest and demand.* GMS J Med Educ. 2025;42(3):Doc33. DOI: 10.3205/zma001757

Dear students,

Gain experience abroad, get to know foreign cultures, make new contacts. There are many good reasons for spending time abroad during your studies. Unfortunately, this has been difficult to realise in dentistry up to now. However, there is now the opportunity to establish co-operations with other universities, for example in Switzerland and Italy.

However, before these collaborations can be intensified, we need your feedback! The following questionnaire asks about your general interest in stays abroad, what kind of stay abroad would be suitable, which countries would be interesting, etc.

The results of this survey form the basis for further action.

Thank you for your participation

IFAS - Department of International Affairs

The Dental Student Council Münster

Part A: Survey exchange dentistry

A1. Gender:

- male ☐
- female ☐
- diverse ☐

A2. Age: ____________

A3. Semester: ____________

A4. **Would you like to complete a semester, internship or clinical traineeship abroad as part of your dental degree programme?**

- Yes ☐
- No ☐

A5. **Would you be prepared to spend a semester abroad if your achievements were not or only partially recognized in Münster?**

- Yes ☐
- No ☐

A6. **What would you prefer? (MC)**

- Erasmus (study visit of at least 1 semester) ☐
- Clinical traineeship (short stay) ☐

A7. **When would you like to go abroad? (MC)**

- 7. Semester ☐
- 8. Semester ☐
- 9. Semester ☐

A8. **I have a language certificate (B2 or higher) in one or more of the following languages:**

(Note: Valid proof must be submitted as part of an application)

- English ☐
- Spanish ☐
- Italian ☐
- Dutch ☐
- French ☐
- Finnish ☐
- Hungarian ☐
- Turkish ☐
- More ☐

A9. **Which partner universities would interest you? (MC)**

- Oulu, Finland ☐
- Basel,Switzerland ☐
- Padua, Italy ☐

Pisa, Italy ☐

Bari, Italy ☐

Riga, Latvia ☐

Coimbra, Portugal ☐

Bratislava, Slovakia ☐

Kosice, Slovakia ☐

Adana, Turkey ☐

Pecs, Hungary ☐

Ghent, Belgium ☐

Leuven, Belgium ☐

Leiden, Netherlands ☐

Spain ☐

France ☐

Japan (traineeship) ☐

USA (traineeship) ☐

A10. In which specialised departments would you like to spend your stay abroad? (MC)

Orthodontics ☐

Oral surgery ☐

Prosthetics ☐

Operative dentistry ☐

A11. What factors would prevent you from travelling abroad? (MC)

Financing ☐

Comment

Loss of time during studies ☐

Comment

Comment

Elaborate planning

Comment

Family/ relationship

Comment

Other

A12. How high would your budget be for a stay abroad?

Thank you for your participation!
After analysing the results, we will inform you about the most important findings.
