# Supplementary material for: International study opportunities in the dentistry degree programme at the University of Münster – a needs assessment of student interest and demand
Source: GMS J Med Educ. 2025 Jun 16;42(3):Doc33. doi: 10.3205/zma001757 (PMC12286880; doi:10.3205/zma001757)
Supplement: Overall evaluation of the “dentistry exchange survey” [file JME-42-33-s-002.pdf]

Attachment 2: Overall evaluation of the "dentistry exchange survey"

Question-ID	Questions with possible answers	Results
A1	Gender:	[%]
	male	73,12
	female	26,58
	divers	0,29
A2	Age:	[Jahre]
	average	22,17 (SD±3,596)
	male	22,72 (SD±4,1)
	female	21,97 (SD±3,4)
A3	Semester:	[%]
	Participation rate 1st-5th semester	54,80
	Participation rate 6th-10th semester	33,20
A4	Would you like to complete a semester, internship or clinical traineeship abroad as part of your dental degree programme?	[%]
	Yes	96,04
	No	3,96
A5	Would you be prepared to spend a semester abroad if your achievements were not or only partially recognised in Münster?	[%]
	yes	54,43
	no	45,57
A6	When would you like to go abroad? (MC)	[%]
	Erasmus (study visit of at least 1 semester)	32,08
	Clinical traineeship (short stay)	29,56
	both	27,49
A7	When would you like to go abroad? (MC)	[%]
	7th. Semester	36,71
	8th. Semester	41,04
	9th. Semester	22,24
A8	I have a language certificate (B2 or higher) in one or more of the following languages:	[%]
	English	85,89
	Spanish	13,51
	Italian	2,70
	Dutch	2,40
	French	23,72
	Finnish	0,00
	Hungarian	0,00
	Turkish	2,70
	More	9,01

Attachment 2 to Hettkamp J, Becker JC, Scherzer S, Marschall B, Ehmke B, Scheutzel P, Junga A. *International study opportunities in the dentistry degree programme at the University of Münster – a needs assessment of student interest and demand.* GMS J Med Educ. 2025;42(3):Doc33. DOI: 10.3205/zma001757

A9	Which partner universities would interest you? (multiple answers possible)	[%]
	Oulu, Finland	59,19
	Basel, Switzerland	58,26
	Padua, Italy	57,01
	Pisa, Italy	65,42
	Bari, Italy	57,63
	Riga, Latvia	30,53
	Coimbra, Portugal	49,84
	Bratislava, Slovakia	12,46
	Kosice, Slovakia	9,35
	Adana, Turkey	12,46
	Pecs, Hungary	13,71
	Gent, Belgium	30,53
	Leuven, Belgium	26,48
	Leiden, Nehterlands	32,09
	Spain	66,98
	France	54,83
	Japan (traineeship)	50,15
	USA (traineeship)	76,01
A10	In which specialised departments would you like to spend your stay abroad? (MC)	[%]
	Orthodontics	24,77
	Prosthetics	23,63
	Operative dentistry	27,17
	Oral surgery	24,43
A11	What factors would prevent you from travelling abroad? (MC)	[%]
	Financing	53,04
	Loss of time during studies	65,81
	Elaborate planing	30,35
	Family/ relationship	17,89
	Other	4,15
A12	How high would your budget be for a stay abroad?	[€]
	average	2929,14 (SD±3296,23)
	male	2599,8 (SD±2321,36)
	female	3365,03 (SD±2835,98)
