# Supplementary material for: International study opportunities in the dentistry degree programme at the University of Münster – a needs assessment of student interest and demand
Source: GMS J Med Educ. 2025 Jun 16;42(3):Doc33. doi: 10.3205/zma001757 (PMC12286880; doi:10.3205/zma001757)
Supplement: Individual responses to the question: What factors would prevent you from going abroad? [file JME-42-33-s-003.pdf]

## Attachment 3: Individual responses to the question: What factors would prevent you from going abroad? (multiple answers possible)

| Question-ID | Individual answers to the question: What factors would prevent you from going abroad? (multiple answers possible) |
|---|---|
| 9 | If it is too expensive, that would be a co-criterion |
| 12 | Can this be combined with Bafög? |
| 15 | Possible scholarships? |
| 24 | Living costs both in Münster and abroad must be covered |
| 39 | Uncertainties regarding financing if you have to finance everything yourself and it is not clear in advance what costs you will incur. |
| 44 | Living costs are higher in some countries, the semester would be additional (if it were not recognised) |
| 50 | Funding similar to Erasmus would be a prerequisite |
| 51 | Support would be necessary |
| 61 | If too expensive in relation |
| 66 | Difficult without Erasmus |
| 107 | Poor financial situation |
| 122 | If the financial situation does not allow it at the moment, then it just won't work |
| 133 | Must not be too expensive |
| 135 | It should be financed at least in part or in total by the university or a partner organisation. |
| 139 | Full financing out of my own pocket would be too expensive for me |
| 142 | Studying is expensive anyway |
| 144 | Unsure |
| 146 | Any materials needed for courses should be provided (you already have them in Münster and can’t take them with you) |
| 168 | Too expensive |
| 194 | Financial support for long stays would be great |
| 195 | Could be expensive and there are no financing options |
| 206 | Often high costs for studying abroad |
| 207 | Scholarships would be good |
| 220 | If high costs have to be borne by students themselves and are not covered as with Erasmus. |
| 224 | Grants e.g. Erasmus |
| 228 | Loans would be necessary |
| 291 | Yes |
| 303 | Costs are too high during or for the stay |
| 318 | Studying is already very expensive anyway |
| 321 | If it becomes too expensive |
| 336 | If the semester is not financed by the university |
| 346 | Tuition fees |
| 3 | You don't want to lose touch |
| 7 | Courses should be recognised by the university |

Attachment 3 to Hettkamp J, Becker JC, Scherzer S, Marschall B, Ehmke B, Scheutzel P, Junga A. *International study opportunities in the dentistry degree programme at the University of Münster – a needs assessment of student interest and demand.* GMS J Med Educ. 2025;42(3):Doc33. DOI: 10.3205/zma001757

| Question-ID | Individual answers to the question: What factors would prevent you from going abroad? (multiple answers possible) |
|---|---|
| 9 | I don’t think I’m prepared to lose a semester, as I've already studied abroad for a year and, at 23, I’m only in my 5th semester |
| 12 | If the work is not recognised at all |
| 19 | As course content is not recognised and the degree programme was not designed for this |
| 51 | You often can’t have anything recognised |
| 66 | Recognition would be very helpful |
| 104 | Non-recognised work and the associated loss of time would be a heavy burden, both financially and socially, as the cohort would be changed. |
| 114 | If credits are not recognised |
| 116 | Unpleasant, but you could live with it |
| 121 | If you lose touch with your cohort as a result |
| 130 | It would be important to me that the standard period of study can be adhered to and that credits earned are recognised |
| 133 | Must not be too high |
| 134 | Recognition of credits in Münster? |
| 143 | Should be recognised |
| 157 | Therefore only clinical traineeships |
| 168 | Additional semesters must be added |
| 192 | Particularly in the case of unrecognised achievements |
| 206 | Lack of recognition of equivalent courses |
| 220 | But only in the sense that hospital partnerships cannot continue. |
| 224 | No more than one semester lost |
| 227 | I would hate to lose a semester, it wouldn’t be worth it for a stay abroad |
| 294 | if you lose a semester as a result |
| 303 | If this makes it impossible to complete the standard period of study |
| 321 | I would hate to lose a semester, but if there’s no other way, one semester isn’t bad either |
| 322 | Exceeding the standard period of study |
| 336 | if we have to repeat the semester in Münster |
| 24 | Time factor in addition to university and free time, which is already limited in itself |
| 44 | Little information, you have to do a lot of research |
| 51 | Signposts for the right approach would be good |
| 59 | Application etc |
| 191 | If you have to take care of everything yourself like at the moment. That’s one of the reasons why I didn’t do it. |
| 206 | Previously time-consuming planning on your own |
| 207 | Support from the university with planning would be great |
| 232 | And bureaucratic hurdles |
| 283 | If you had to plan everything yourself |
| 294 | If it's too time-consuming and not related |
| 318 | You don’t have much time for other things during the semester |

| Question-ID | Individual answers to the question: What factors would prevent you from going abroad? (multiple answers possible) |
|---|---|
| 328 | if not recognised, finding accommodation, local contact |
| 349 | Complicated application documents |
| 12 | at most one semester |
| 24 | Therefore only a clinical traineeship instead of a whole semester |
| 45 | Long distance from spouse |
| 195 | Friend in DE |
| 200 | Friend is at home |
| 216 | Partner and 1 child (school-age) are alone |
| 291 | Yes |
| 362 | Friend |
| 12 | Language barrier: Only with English teaching format |
| 53 | If I was the only one of my entire circle of friends who had to repeat a semester in Münster because of a semester abroad, I wouldn’t do it |
| 63 | Travel restrictions |
| 141 | None of the above, as long as it doesn't go completely beyond the scope |
| 222 | Entry restrictions |
| 279 | Language skills |
| 303 | Very poor facilities at the destinations |
| 306 | Travel restrictions |
| 328 | No reference persons during the semester |

1
